# The Periparturient Gut Microbiota’s Modifications in Shaziling Sows concerning Bile Acids

**DOI:** 10.3390/metabo13010068

**Published:** 2023-01-01

**Authors:** Jie Wang, Yulian Li, Chang Cao, Runhua Yang, Meilin He, Jiaqi Yan, Peng Huang, Bie Tan, Zhiyong Fan

**Affiliations:** 1 College of Animal Science and Technology, Hunan Agricultural University, Changsha 410128, China; 2 Xiangtan Livestock Breeding Station, Xiangtan Agriculture and Rural Bureau, Xiangtan 411100, China

**Keywords:** Shaziling pig, perinatal period, gut microbiota, bile acid

## Abstract

Shaziling pigs, as a native Chinese breed, have been classified as a fatty liver model. As the core of the whole pig farm, the sow’s organism health is especially important, especially in the perinatal period; however, there are few reports on the perinatal intestinal microbiology and bile acid metabolism of Shaziling pig sows. The purpose of this research was to investigate the alterations in bile acids and gut microbiota of sows that occur throughout the perinatal period. Forty-two sows were selected for their uniformity of body conditions and were given the same diet. Fecal samples were collected for 16srDNA sequencing and bile acid targeted metabolome detection in four stages (3 days before delivery, 3 days after delivery, 7 days after delivery and 21 days after delivery). As revealed by the results, there were statistically significant variations in bile acids among the four stages, with the concentration of bile acids identified by SZL-4 being substantially greater than that of the other three groups (*p* < 0.05). When compared to the other three groups (*p* < 0.05), SZL-2 had considerably lower Shannon, Simpson and Chao 1 indices, and exhibited a statistically significant difference in β-diversity. SZL-2 samples included a greater proportion of *Proteobacteria* than SZL-3 and SZL-4 samples; however, SZL-2 samples contained a smaller proportion of spirochetes than SZL-3 and SZL-4 samples. To a large extent, lactic acid bacteria predominated in the SZL-2 samples. The LEfSe analysis showed that the relative abundances of *Lachnospiraceae_XPB1014_group*, *Christensenellaceae_R_7_group*, *Clostridium, Collinsella, Turicibacter*, and *Mollicutes_RF39_unclassified* were the main differential bacteria in the SZL-1 swine fecal samples and the *Eubacterium__coprostanoligenes_group* in sow fecal samples from SZL-2. The relative abundance of *Bacteroides, UBA1819, Enterococcus, Erysipelatoclostridium*, and *Butyricimonas* in SZL-3 and SZL-4 *Streptococcus, Coriobacteriaceae_unclassified, Prevotellaceae_UCG_001, Streptomyces*, and *Ochrobactrum* in SZL-3. g_*Collinsella* was significantly and positively correlated with vast majority bile acids, and the *g_Lachnospiraceae_XPB1014_group* with GCDCA and GHDCA into positive correlations. Simultaneously, *g_Streptococcus*, *g_Bacteroides*, and *g_UBA1819* inversely correlated with bile acid, accounting for the great bulk of the difference. In conclusion, there is an evident correlation between bile acids and gut microbiota in the perinatal period of Shaziling sows. Additionally, the discovery of distinct bacteria associated to lipid metabolism gives a reference for ameliorating perinatal body lipid metabolism disorder of sows through gut microbiota.

## 1. Introduction

The periparturient period of a sow is the transition period from gestation to lactation, and is typically comprised of late gestation and early lactation. In fact, sows are the backbone of current large-scale pig production; the health and reproductive success of breeding sows is crucial to the economic efficiency of pig farms [1]. Multiple studies have demonstrated that sow gut microbiota and bile acid metabolic equilibrium are critical for the intestinal development of periparturient offspring [2,3,4]. Sows have higher systemic oxidative stress during the periparturient period because of the increased metabolic load [5,6]. Overloading the metabolic processes promotes insulin sensitivity to decline just before birth, whereas insulin resistance gradually increases [7]. Compared with obesity, which is detrimental to health, decreased insulin sensitivity is beneficial for fetal growth and energy requirements during lactation, but in sows, excessive decreased insulin sensitivity during the perinatal period leads to decreased feeding during lactation [8]. Future researchers will concentrate on identifying solutions to the perinatal metabolic syndrome in sows.

By promoting the absorption of nutrient uptake from ingested food and producing a vast variety of metabolites for the organism, the gut microbiota makes a substantial contribution to maintaining internal environmental homeostasis [9]. Prior to and after parturition, hormonal, metabolic, and immunological variables all influence the composition of the sow’s gut microbiota. Researchers have shown that although pregnant women’s gut microbiota are comparable to those of healthy controls in the first trimester, the constitution of the gut flora system changes substantially by the third trimester, with a significant increase in gut flora diversity. Furthermore, evidence suggests that the composition of the gut microbiota influences immunological development, pathogen protection, and the occurrence of a variety of chronic disorders [10]. Following their production in the liver from cholesterol, bile acids undergo further processing by the intestinal microbiota in the gut [9]. There is a substantial correlation between bile acid metabolism and obesity and metabolic problems [11]. Wang et al. discovered that fasting total bile acid levels in T2DM patients were associated with decreased insulin sensitivity, poor islet cell function, and abnormally high glucagon levels at high normal values, implying that increased fasting total bile acid levels may harm islet cell and cell secretion, and that elevated fasting total bile acid levels may be the cause of some chronic diabetic complications [12]. Bile acids (BAs) are antimicrobial compounds with the potential to affect the gut microbial ecology, as has been established in a number of studies. This is because the gut microbiota maintains bile acid homeostasis via complex interactions. Conversely, BA microbial metabolism broadens the diversity of the BA pool [13,14,15]. Henceforth, sows with perinatal metabolic abnormalities and insulin resistance might benefit from a novel method that involves investigating the connection between gut microbes and bile acids.

The Shaziling pig, a native breed in China, has been listed as a fatty liver model [16]. In comparison to imported pig breeds, it has greater amounts of intramuscular fat and better quality meat. Previous studies have mainly discussed the genetic differences between Shaziling pig breeds and introduced pig breeds, including epigenetics, quantitative genetics, and metagenomics [17,18]. The in-depth research and promotion of the Shaziling pig is hindered, however, by the insufficient research on the changes in intestinal microbial metabolites throughout the perinatal period. Additional research on lipid metabolism and insulin resistance in Shaziling pigs, a model of fatty liver disease, is necessary. Therapeutically, bile acids have been demonstrated to improve symptoms of non-alcoholic fatty liver disease (NAFLD) by activating on certain receptors. Recent research in mice and humans has shown that regulating bile acids and receptors can improve insulin secretion and sensitivity, suppress lipogenesis, and stimulate FAs oxidation [19].

In this study, using local fat pigs (Shaziling pigs), a total of four-time points before and after parturition were selected to compare the community diversity and structure of the gut microbiota, as well as the bile acid composition and the correlation between the two using 16S rRNA gene sequencing and bile acid-targeted metabolomics in the four perinatal stages of Shaziling pigs. The overarching purpose of this research is to compare and contrast the kinds of bacteria and bile acids present throughout pregnancy at various stages, as well as to identify any trends or correlations that may emerge. As a result, it is a valuable resource for advancing breeding practices by providing a benchmark for working with Shaziling sows.

## 2. Materials and Methods

### 2.1. Animal, Diets and Experimental Design

The animal feeding was carried out on a commercial pig farm. Fifty-four healthy, similar weight, similar body condition 3-4 litters Shaziling pregnant sows. Feces samples were collected 3 days before delivery (SZL-1), 3 days after delivery (SZL-2), 7 days after delivery (SZL-3), and 21 days after delivery (SZL-4) for 16S rDNA sequencing and cholic acid target detection. The composition of the basal diet (Table S1) was based on the Nutritional Needs of Chinese Pigs. Sows were housed in 2.0 m by 0.6 m concrete floor enclosures from day 85 to day 110 of gestation. From day 110 of gestation to weaning, sows were kept in indoor 2.13 m by 0.66 m concrete-floor farrowing enclosures. The sows and piglets were then individually housed in farrowing pens with crates, slatted floors, and heating pads for the piglets. The sows and piglets had free access to water. The experiment was carried out at the Shaziling Resource Farm in Xiangtan, Hunan Province, and the feeding management and immunization procedures were carried out by the company’s standard breeding management.

### 2.2. Sample Collection

Fresh fecal samples were collected at the same time point (17:00) from each sow 3 days before parturition, 3 days after parturition, 14 days before weaning, and on the day of weaning. Before collecting feces, the sows were washed with potassium permanganate solution to clean the dirt on the rump, then disinfected with alcohol cotton balls. Disposable gloves were used to apply lubricating oil and around 2 g feces was taken rectally, loaded into fecal collection tubes, and put into a −20 °C refrigerator for temporary storage.

### 2.3. Sample Preparation and HPLC-MS/MS Analysis

Thirty milligrams was weighed from fecal samples of sows (3 days before delivery, 3 days after delivery, 7 days after delivery and 21 days after delivery), and then 100μL of pre-cooled ultrapure water added for homogenization (using the instrument: MP Fastprep-24 Automated Homogenizer (MP Biomedicals), 500 μL of pre-cooled methanol and 10 μL of internal standard added, and then samples were vortexed and mixed, incubated at −20 °C for 20 min to precipitate the protein, centrifuged at 14,000× *g* rcf for 15 min at 4 °C, and the supernatant vacuum dried. For mass spectrometry, methanol-water (1:1, *v*/*v*) was added and re-solubilized, the samples were centrifuged at 14,000× *g* rcf for 15 min at 4 °C, and the supernatant was taken into the sample for analysis. Samples were separated using a Waters ACQUITY UPLC I-Class system. The mobile phase contained A = 0.1% formic acid aqueous solution (Honeywell, 94,318) and B = methanol (Fisher Chemical, A452-4). The samples were in the automatic sampler at 8 °C, and the column temperatures were kept constant at 45 °C. The gradients were at a flow rate of 300 uL/min, and a 2 µL aliquot of each sample was injected. The gradient was 60% B, which linearly changed to 65% in 0–6 min, and then changed to 80% in 6–13 min and changed to 90% in 13–13.5 min; then, the B was maintained for 13.5–15 min. A quality control (QC) was set for each number of samples in the sample cohort to detect and evaluate the stability and reproducibility of the system. Mass spectrometry was performed using a 5500 QTRAP mass spectrometer (AB SCIEX) in negative ion mode, 5500 QTRAP ESI. The source conditions were as follows: source temperature: 550 °C; ion Source Gas1 (Gas1): 55; Ion Source Gas2 (Gas2): 55; Curtain gas (CUR): 40; ionSapary Voltage Floating (ISVF): −4500 V. The ion pairs were detected in MRM mode and the peak areas and retention times were extracted using Multiquant software. The retention time was corrected for metabolite identification using a standard for bile acids. All samples were mixed in equal amounts and prepared as QC samples. The stability and reproducibility of the data were evaluated by using QC samples, and the relative standard deviation (RSD) of metabolites were less than 30%. The regression fitting correlation coefficients (R2) of all analytes were above 0.99. The analytical indexes indicated that the method could accurately quantify the target metabolite concentrations in the colon contents within the above concentration range.

### 2.4. DNA Extraction, 16S rDNA Amplification, and 16S rRNA Sequencing

DNA was extracted from fecal samples from sows (3 days before delivery, 3 days after delivery, 7 days after delivery, and 21 days after delivery) using the E.Z.N.A. ^®^Stool DNA Kit (D4015, Omega, Inc., Norwalk, CT, USA). The V3–V4 region of the bacterial 16S rRNA gene was amplified with slightly modified versions of primers 338F (5′-ACTCCTACGGGAGGCAGCAG-3′) and 806R (5′-GGACTACHVGGGTWTCTAAT-3′). Bacterial 16S rRNA genes were amplified by PCR. The amplicon pools were prepared for sequencing and the size and quantity of the amplicon library were assessed on Agilent 2100 Bioanalyzer (Agilent, Santa Clara, CA, USA) and with the Library Quantification Kit for Illumina (Kapa Biosciences, Woburn, MA, USA), respectively. The PhiX Control library (v3) (Illumina) was combined with the amplicon library (expected at 30%). The libraries were sequenced in a 300PE MiSeq run, and one library was sequenced using standard Illumina sequencing primers using both protocols. Samples were sequenced on an Illumina MiSeq platform according to the manufacturer’s recommendations, provided by LC-Bio. Paired-end reads were assigned to samples based on their unique barcode and truncated by cutting off the barcode and primer sequence. Paired-end reads were merged using FLASH. Quality filtering on the raw tags was performed under specific filtering conditions to obtain high-quality clean tags according to the FastQC (V 0.10.1). Sequences with ≥97% similarity were assigned to the same operational taxonomic unit (OTU) using Verseach (v2.3.4). Representative sequences were chosen for each OTU, and taxonomic data were then assigned to each representative sequence using the RDP (Ribosomal Database Project) classifier. The differences of the dominant species in different groups and multiple sequence alignment were conducted using the PyNAST software to study the phylogenetic relationship of different OTUs. OTUs abundance information was normalized using a standard sequence number corresponding to the sample with the least sequences. Alpha diversity was analysed by four indices to analyse the complexity of species diversity in the samples, including Chao1, Shannon, Simpson and Observed species. All these indices in our samples were calculated using QIIME (version 1.8.0). Beta diversity was analysed by principle coordinate analysis (PCoA) and QIIME software (version 1.8.0) for cluster analysis. Significant differences in α-diversity and the number of OTUs between groups were determined by one-way variance scores (ANOVA) and Duncan’s multiple comparison test using SPSS software (SPSS statistics 20).

### 2.5. Statistical Analysis

Statistical analysis was performed using SPSS 20.0 software (SPSS Inc., Chicago, IL, USA). The bile acid content and flora abundance between the groups were statistically analyzed, and then Duncan multiple comparisons were performed. When the data did not conform to normal distribution or homogeneity, the Kruskal–Wallis test was used. The results are expressed as mean ± standard error (SEM). *p* < 0.05 is considered significant, 0.05 ≤ *p* ≤ 0.10 is considered a trend. GraphPad Prism version 8.0.0 for Windows (GraphPad Software, San Diego, CA, USA) was used to plot the data after SPSS analysis. Bioinformatic analysis was performed using the OmicStudio tools at https://www.omicstudio.cn/tool (accessed on 7 October 2022). Principal component analysis (PCA) was used to distinguish the metabolic profiles among the groups. Based on the results of Pearson correlation analysis, a correlation heat map of bile acids and intestinal flora was generated.

## 3. Results

### 3.1. Changes in Bile Acids in the Feces of Shaziling Sows Periparturient

**Figure 1:**
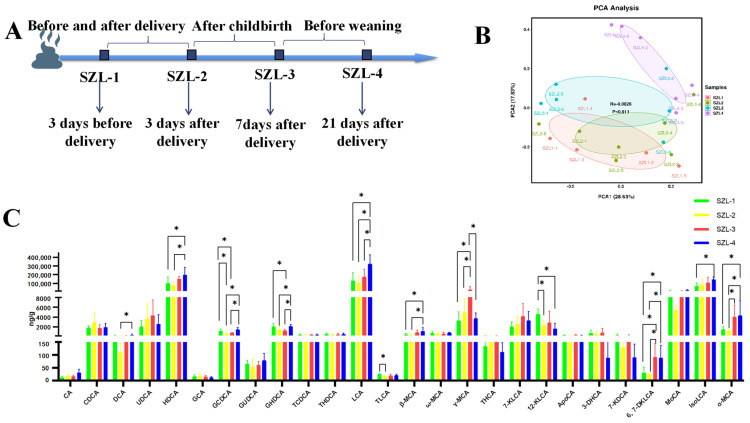
Changes in bile acids in the feces of Shaziling sows periparturient. (**A**) Fecal sample collection time point. (**B**) Bile acid principal coordinates (PCA) analysis between the four groups. (**C**) The status of differences between the four groups of bile acids. Data are presented as means + SEM (*n* = 5−6/group). * *p* < 0.05.

Sows were tested for the presence of 26 bile acids in the feces using a bile acid targeting metabolite to learn more about the variation of gut bile acids in SZL-1 to SZL-4. This variation in bile acid composition between SZL-4 and the other three groups was further elucidated by a later principal coordinates analysis (Figure 1B). The differences in bile acids between groups were analyzed by one-way ANOVA for each of the 26 bile acids between groups showed that deoxycholic acid (DCA), hyodeoxycholic acid (HDCA), glycochenodeoxycholic acid (GCDCA), β-Muricholic acid (β-MCA), glycohyodeoxycholic acid (GHDCA), lithocholic acid (LCA), taurolithocholic acid (TLCA), γ-muricholic acid, (γ-MCA), 12-ketolithocholic acid (12-KLCA), 6,7-diketolithocholic acid (6,7-DKLCA), isolithocholic acid (IsoLCA), and α-muricholic acid (α-MCA) were significantly different between groups; notably, DCA, HDCA, GCDCA, β-MCA, LCA, GHDCA, IsoLCA, and α- MCA were highest in the SZL-4 phase and differed more significantly from the other groups (Figure 1C). 

To further investigate the changes in bile acids during the SZL-4 phase, a two-by-two comparison of SZL-4 with the other phases revealed that the concentrations of cholic acid (CA) and 6,7-DKLCA were significantly higher in SZL-4 than in SZL-1 and SZL-2 (*p* < 0.05, Figure S1A,B,K,L). Furthermore, the concentrations of LCA were highly significantly higher than in SZL-1 and SZL-2 (*p*< 0.01, Figure S1E,F), and significantly higher than SZL-3 (*p* < 0.05, Figure S1G). Substantially greater levels of HDCA, β-MCA, ω-muricholic acid (ω-MCA), murocholic acid (MoCA), and α-MCA were discovered in SZL-4 compared to SZL-2 (*p* < 0.05, Figure S1C,H,I,N,O), whereas IsoLCA concentrations were highly significantly higher in SZL-4 than in SZL-1 (*p* < 0.01, Figure S1N). GHDCA concentrations were highly significant higher in SZL-4 than in SZL-3 (*p* < 0.01, Figure S1D), and notably, only 12-ketolithocholic acid (12-KLCA) concentrations were significantly lower and highly significantly lower than in SZL-1 (*p* < 0.01, Figure S1J).

### 3.2. Changes in Intestinal Microorganisms in the Feces of Shaziling Sows Periparturient

**Figure 2:**
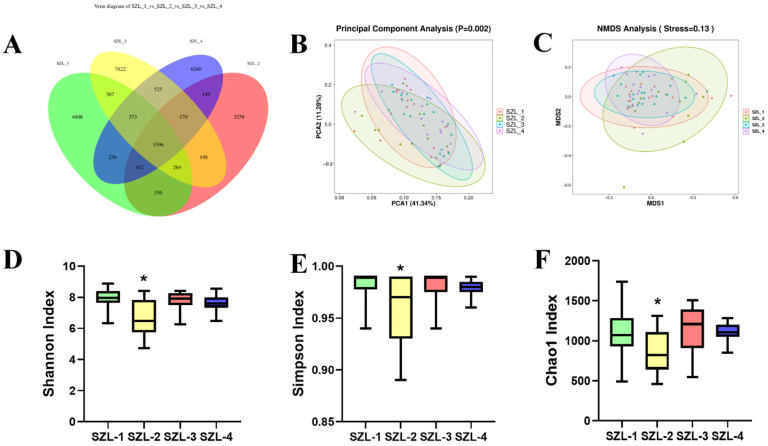
Changes in gut microbiome of Shaziling sows periparturient. (**A**) The OTUs in a Venn diagram. (**B**) The β−diversity of the microbial community expressed as PCA. (**C**) NMDS analysis. (**D**) The α-diversity of the Shannon index, (**E**) Simpson index, and (**F**) Chao 1 index of fecal microbes. “*” represent significant difference, *p* < 0.05.

Fecal samples collected from Shaziling sows at SZL-1, SZL-2, SZL-3, and SZL-4 had OTUs of 8182, 6153, 11,335, and 9491, respectively; whereas, 1596 common OTUs were identified (Figure 2A). 16S rDNA sequencing was used to investigate the composition of the gut microbiota at various stages. In all samples, the test analyzed the para-colonial nature of the microbial community and performed PCA analysis (*p* < 0.05, Figure 2B,C). Microbial community characteristics varied widely across SZL-1, SZL-2, SZL-3, and SZL-4, with more clustering occurring among SZL-1, SZL-3, and SZL-4. The results of NMDS were identical to those of PCA, with the exception that clustering between SZL-2 and the remaining three groups was more dispersed. The microbiota diversity, as measured by the Shannon index, Simpson index, and Chao 1 index, is shown in Figure 2D–F. Fecal samples from SZL-2 had a lower Shannon index, Simpson index, and Chao 1 index than those from other ages (*p* < 0.05). From the α-diversity and β-diversity, it is apparent that the huge disparity in richness between the SZL-2 group and the remaining groups’ microbial diversity may be attributable to the influence of parturition on the Shaziling sows, resulting in a decrease in the richness and diversity of the gut microbiota. 

**Figure 3:**
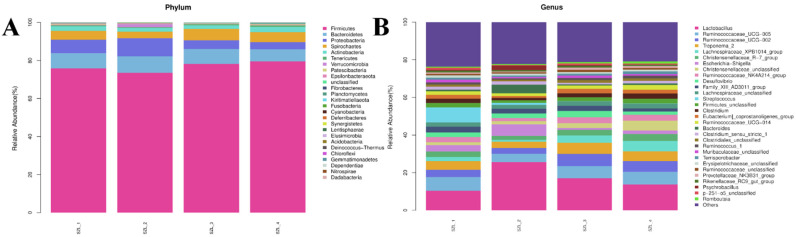
Changes in the structure of the microbial community of Shaziling sows periparturient. (**A**) The relative abundance of bacterial compositions at the phyla level of the bacteria in the feces sample. (**B**) The relative abundance of horizontal bacterial compositions at the genera level in the feces sample.

In terms of bacterial phylum, approximately 85% of the total bacteria were classified as belonging to the *Firmicutes* and *Bacteroidetes* (Figure 3A). The relative content of the *Proteobacteria* phylum was higher in SZL-2 samples than in SZL-3 and SZL-4, while the relative content of *Spirochaetes* phylum was lower than in the other three groups. At the genus level, the relative abundance of *Lactobacillus* was higher and dominated in samples from SZL-2 (Figure 3B). However, at subsequent points, the relative abundance of *Lactobacillus* was significantly lower than that of SZL-2 (Figure 3B), while the relative abundance of *Ruminococcacae-UCG-005* was dramatically lower than the other three time points (Figure 3B).

### 3.3. Analysis of Gut Microbiota Differential Bacteria and Their Correlation with Bile Acids in the Feces of Shaziling Sows Periparturient

**Figure 4:**
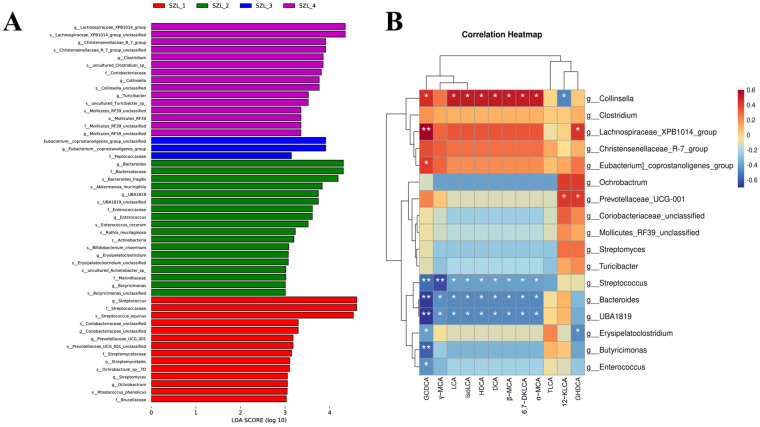
Analysis of gut microbiota differential bacteria and their correlation with bile acids in the feces of Shaziling sows periparturient. (**A**) The differences in bacteria composition at different time points. (**B**) Analysis of Spearman’s correlation between significantly altered BAs and microbiota. “*” and “**” represent significant difference, *p <* 0.05 and *p <* 0.01, respectively.

Additional study of the microbial compositions throughout the four phases of the breeding stages were performed using the linear discriminant analysis effect size (LefSe) (Figure 4). Our LEfSe analysis revealed that at the genus level, the cladogram showed that the relative abundances of *g_Lachnospiraceae_XPB1014_group*, *g_Christensenellaceae_R_7_group*, *g_Clostridium*, *g_Collinsella*, *g_Turicibacter* and *g_Mollicutes_RF39_unclassified* were higher in the pig fecal samples from SZL-1, and *g_Eubacterium__coprostanoligenes_group* in fecal samples of sows from SZL-2. Our research revealed that *g_Bacteroides*, *g_UBA1819*, *g_Enterococcus*, *g_Erysipelatoclostridium* and *g_Butyricimonas* were present in SZL-3 and *Streptococcus*, *g_Coriobacteriaceae_unclassified*, *g_Prevotellaceae_UCG_001*, *g_Streptomyces* and *g_Ochrobactrum* were present in SZL-4 (Figure 4A and Figure S2).

Figure 4B demonstrates that the quantity of BAs in feces is proportional to the bacterial diversity present. We determined that the *g_Collinsella* associated with bile acid into the vast majority of differences, and the *g_Lachnospiraceae_XPB1014_group* with GCDCA and GHDCA into positive correlations. Concurrently, the bulk of the disparity can be attributed to the negative correlation between bile acid and *g_Streptococcus*, *g_Bacteroides*, and *g_UBA1819*. Furthermore, the relative abundance of the primary cholesterol-degrading bacteria was compared to reflect the influence on the microbiome before and after delivery (Figure S3A–F). It was discovered that *g_Lactobacillus, g_Bacteroides*, *g_Enterococcus*, and *g_Bifidobacterium* had the highest abundance at SZL-2, *g_Parabacteroides* at SZL-3, and *g_Akkermansia* before parturition, which diminished progressively with parturition and recovered at weaning.

## 4. Discussion

During pregnancy, a woman’s body undergoes drastic changes that require each organ to be adjusted for optimal functioning. After giving birth, the woman must breastfeed her baby, leading to significant energy consumption. Changing glucose metabolism is a hallmark of the metabolic adjustments that women undergo throughout their pregnancies to meet the demands of their developing babies. Throughout pregnancy, insulin sensitivity, pancreatic cell function, and hepatic gluconeogenesis are altered, which facilitates the shunting of glucose and nutrients to the developing fetus [20]. Over the last few decades, evidence has accumulated suggesting that bile acids play an increasingly significant function as signalling molecules in metabolic regulation, which is especially pertinent to pregnancy [3,21]. In order to address these metabolic issues in sows, it is important to understand how bile acids fluctuate and behave throughout pregnancy. Research indicates that stages SZL-1, SZL-2, and SZL-3 have significantly different bile acid compositions than stage SZL-4. The most striking difference was that SZL-4 contained significantly more differential bile acids than the other three groups did, with the exception of γ-MCA and 12-KLCA. A subsequent two-by-two comparison of the SZL-4 group with the remaining three groups revealed that HDCA, LCA, β-MCA, ω- MCA, MoCA, GHDCA, IsoLCA, and secondary bile acids were higher than the rest of the groups, thus indicating that the conversion of primary to secondary bile acids in the intestine was the greatest, suggesting the strongest modification of bile acids by intestinal microorganisms. It has been postulated that the sow’s metabolic problems will be alleviated 21 days after birth, as this is when the gut microbes are most likely to have recovered and reconstruct. Therefore, microbiological investigation of sow feces was performed at four different stages of the intestinal system.

The gut microbiota of sows directly affects the reproductive performance and health of the organism. However, there are few longitudinal studies on the systematic changes of gut microbiota in sows during the perinatal period [22]. This experiment investigated the evolution of gut microbiota and changes in bile acid metabolism in the periparturient period of sows in Shaziling. It has been found that changes in the gut microbial composition may be related to the physiology of pregnancy, during which there are dramatic changes in maternal metabolism, reproductive hormones, and immune function. It has been found that the gut microbial composition is similar in the first trimester and the absence of pregnancy, but changes as the pregnancy progresses [23]. Our analyses revealed that the SZL-2 stage contained the lowest OTUs. Overall, SZL-2 sows exhibited the lowest diversity and richness of gut microbes, as measured by the Chao-1 index, Simpson index, and Shannon index, when compared to the other groups. Our results suggest that the gut flora of sows is altered due to farrowing stress, and that after farrowing, the composition of the gut flora gradually returns to that at farrowing.

More than 90% of the entire sequencing was determined to originate from the thick-walled phylum and the bacteriophage phylum in the pig intestinal microbiota. Although other phyla such as the variola and spirochete phyla made up a small percentage of the community, they made up less than 5% on average. The composition of the thick-walled phylum and the bacteriophage phylum differed between breeds, with Duroc pigs (81.7%, *p* = 0.064), Long White pigs (83.2%, *p* = 0.030), and Great Yorkshire pigs (87.0%, *p* = 0.003) having a significantly higher proportion of thick-walled phylum in their feces than Hampshire pigs (74.2%). In contrast, the proportion of *Bacillus* phylum in the feces of Hampshire pigs (22.2%) was higher than that of Duroc pigs (14.8%, *p* = 0.065), Long White pigs (12.5%, *p* = 0.019), and Yorkshire pigs (9.13%, *p* = 0.002) [24]. As with previous results, species analysis at the phylum level showed that the first, second, and third dominant bacteria in all four stages were *Firmicutes* and *Bacteroidetes*, accounting for more than 80% of the total community, with other subdominant phyla including *Proteobacteria* as well as *Spirochaetes*. Comparing SZL-1 and SZL-2, we discover that SZL-2 has a lower *Firmicutes/Bacteroidetes* ratio; this ratio then gradually increases in SZL-3 and SZL-4, reaching its maximum value in SZL-4. In conclusion, the data presented above indicate that a sow’s organism undergoes shifts before and after parturition, and that an increase in the proportion of Firmicutes to Bacteroidetes after birth may play a role in facilitating the sow’s body’s transition from lipid deposits to lean tissue.

During the perinatal phase, the sow’s gut microbiota is especially vulnerable to disturbances due to oxidative stress spurred on by the rapid changes in the sow’s physiological metabolism and nutritional requirements. At the genus level, SZL-2 sow feces exhibited a greater number of bacteria than the other three groups, with Lactobacillus, a rod-shaped, gram-positive, non-spore-forming, parthenogenic anaerobic ‘thick-walled phylum’, being the first dominant bacterium. *Lactobacillus* can regulate intestinal microbial balance by regulating intestinal pH, secreting antimicrobial peptides, and competing for occupancy [25]. According to research by Wang et al., supplementing sows with *Lactobacillus* boosts their antioxidant levels and reproductive efficiency [26]. Matthew et al. [25] investigated the role of BSHs encoded by *Lactobacillus acidophilus* and found that BA-type and bile salt hydrolases (BSHs) substrate preference affect the growth of *L. acidophilus* in vitro and in vivo, and that *Lactobacillus acidophilus*-encoded BSHs are not used to promote colonization and detoxify bile, but rather their enzymatic preference and the inherent chemical characteristics of BAs determine their toxic effects on bacterial growth; BSHs act as probiotic colonization promoters and intervene to treat disease by lowering serum cholesterol or during obesity [27,28,29]. Therefore, it is worth noting that the increase in fecal *Lactobacillus* abundance during SZL-2 compared to the other three time points may be due to an increase in beneficial bacteria (*Lactobacillus* content) by the Sandy Ridge sow organism to restore the gut microbes to their prepartum state, which may be related to bile acid-related BSHs.

There was a substantial association between bile acid metabolism and intestinal microbes, as determined by correlation analysis of various bacteria and bile acids. Most of the distinctions can be attributed to bile acid and *g_Collinsella* associated with bile acid into the vast majority of differences. Stuart, Luisa et al. [30,31]. found that the abundance of *Collinsella* was positively correlated with fasting triglyceride and total cholesterol concentrations, and negatively correlated with HDL cholesterol concentrations. Additionally, there was a positive correlation between insulin levels and the abundance of *Corynebacterium* spp., suggesting that the metabolism of this flora performs a regulatory effect on the body’s lipid metabolism [31,32]. In addition, we analyzed the cholesterol-degrading flora between the groups and found that the abundance of cholesterol-degrading-related bacteria *g_Lactobacilus, g_Bacteroides, g_Bifidobacterium*, and *g_Enterococcus* was significantly higher in the SZL-2 group than in the other groups. Since there was a larger population of these four species, we reasoned that the sows’ lipolysis and lactation rates would improve and accelerate just before they gave birth.

## 5. Conclusions

To sum up, throughout the periparturient period, Shaziling sows indicated considerable variation in bile acid composition and gut microbiota diversity, with a proven connection between differential bacteria and differential bile acids, and we also screened for *Collinsella*, an uncommon bacterium associated with lipid metabolism and insulin resistance. After discovering a positive correlation between *Collinsella* and the vast majority of differential bile acids, we suggest that the presence of this bacteria may contribute to the development of fatty liver in Shaziling pigs serving as model pigs. This study can provide a reference for researchers to further understand the periparturient physiological metabolic changes and alleviate the metabolic disorders in the sows of Shaziling. Constipation in the sow prevented us from collecting fecal samples on the day of birth, which is a limitation of the research. This investigation on the ramifications of exogenous bile acid on the microbiome of Shaziling sows and on their reproductive performance and lipid metabolism after the bacteria in their gut were given less control over their activity has been shown. As a final recommendation, farmers should first take care of their sows’ intestinal health. Selectively cultivating the optimal active microbiome benefits sows in a number of ways, including improved bowel health of the sows, alleviated metabolic diseases and enhanced reproductive performance.

## Data Availability

The data that support the findings of this study are available from the corresponding author, Zhiyong Fan, upon reasonable request. The data are not publicly available.

## Supplementary Materials

The following supporting information can be downloaded at: https://www.mdpi.com/article/10.3390/metabo13010068/s1, Table S1: Composition and nutrient levels of basal diets.
