# Supplementary material for: The Periparturient Gut Microbiota’s Modifications in Shaziling Sows concerning Bile Acids
Source: Metabolites. 2023 Jan 1;13(1):68. doi: 10.3390/metabo13010068 (PMC9863110; doi:10.3390/metabo13010068)
Supplement: Supplementary file 1 [file metabolites-13-00068-s001.zip › metabolites-2115785-supplementary Table.pdf]

Supplementary Table S1  Composition and nutrient levels of basal diets

| Igredient (%) | Content | |
|---|---|---|
| | Late gestation | lactation |
| Soybean meal | 5.40 | 19.50 |
| Corn | | 43.18 |
| Wheat | 20.00 | 12.00 |
| Rice | 38.98 | 13.00 |
| Bran | 18.20 | 1.00 |
| Rice bran | 9.70 | 4.00 |
| Soybean Oil | 2.00 | 2.00 |
| All premixes | 5.72 | 5.32 |
| Total | 100.00 | 100.00 |
| Nutrient composition | Late gestation | lactation |
| DE MJ/Kg | 13.10 | 14.58 |
| ME MJ/Kg | 12.62 | 14.05 |
| CP % | 12.38 | 15.22 |
| SID Lysine | 0.44 | 0.68 |
| SID Methionine | 0.18 | 0.23 |
| SID Threonine | 0.32 | 0.47 |
| SID Tryptophan | 0.12 | 0.14 |
| SID Valine | 0.54 | 0.65 |
| SID Methionine+Cysteine | 0.33 | 0.41 |
| CF % | 7.37 | 5.10 |
| NDF % | 19.88 | 15.76 |
| Ca % | 0.07 | 0.10 |
| TP % | 0.52 | 0.38 |
