# Supplementary material for: The Periparturient Gut Microbiota’s Modifications in Shaziling Sows concerning Bile Acids
Source: Metabolites. 2023 Jan 1;13(1):68. doi: 10.3390/metabo13010068 (PMC9863110; doi:10.3390/metabo13010068)

Figure S1. Bile acids with significant differences between SZL-4 and SZL-1, SZL-2 and SZL-3 after a two-way comparison.(A-O).“*” and “**” represent signifificant difference, $p < 0.05$ and $p < 0.01$, respectively.

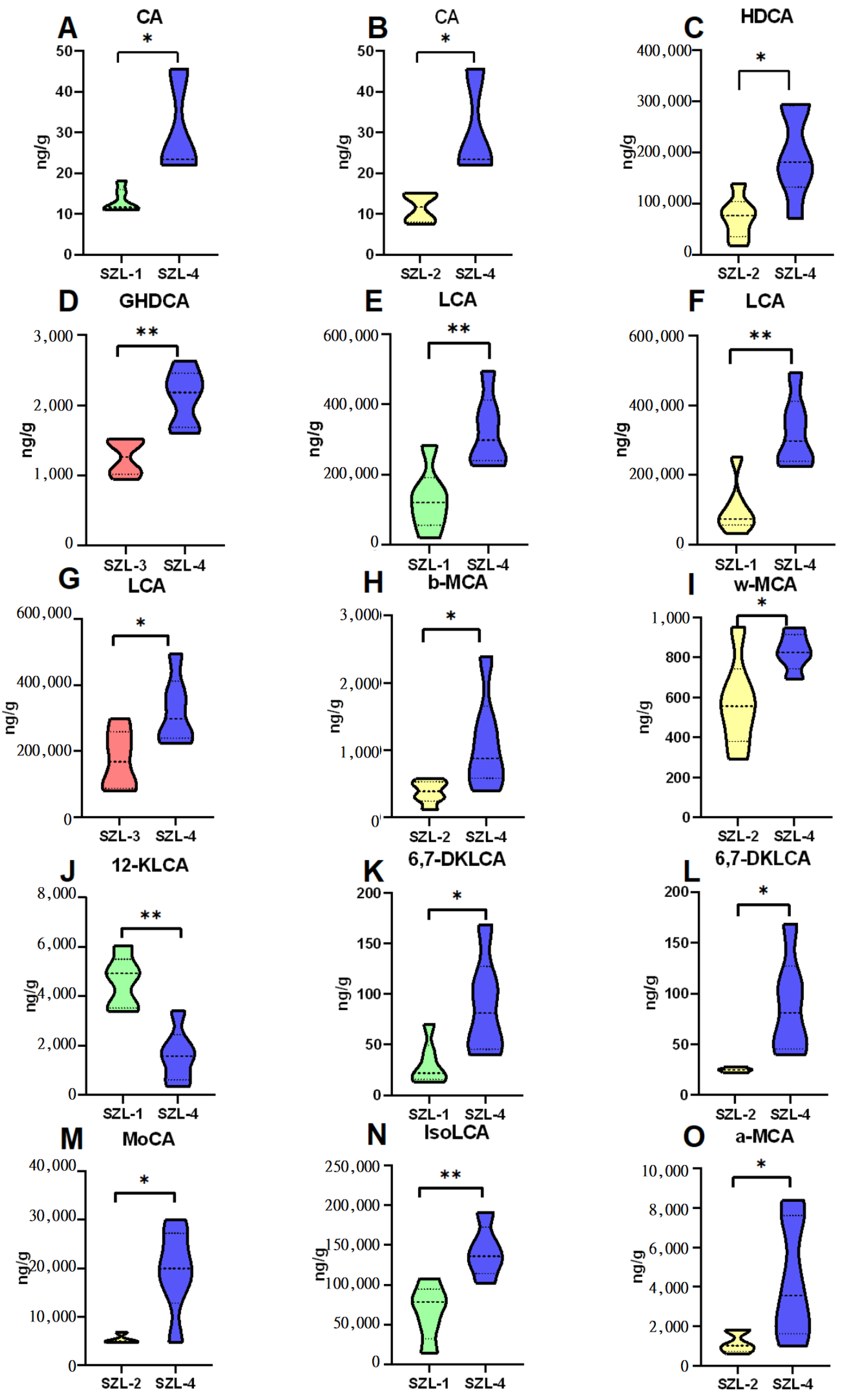

Figure S2. LEfSe analysis of gut microbiota composition throughout the reproductive cycle of the sow. Clade map using the LEfSe method showing the phylogenetic distribution of the sow's gut microbiota throughout the reproductive cycle. Each successive circle represents a phylogenetic level.

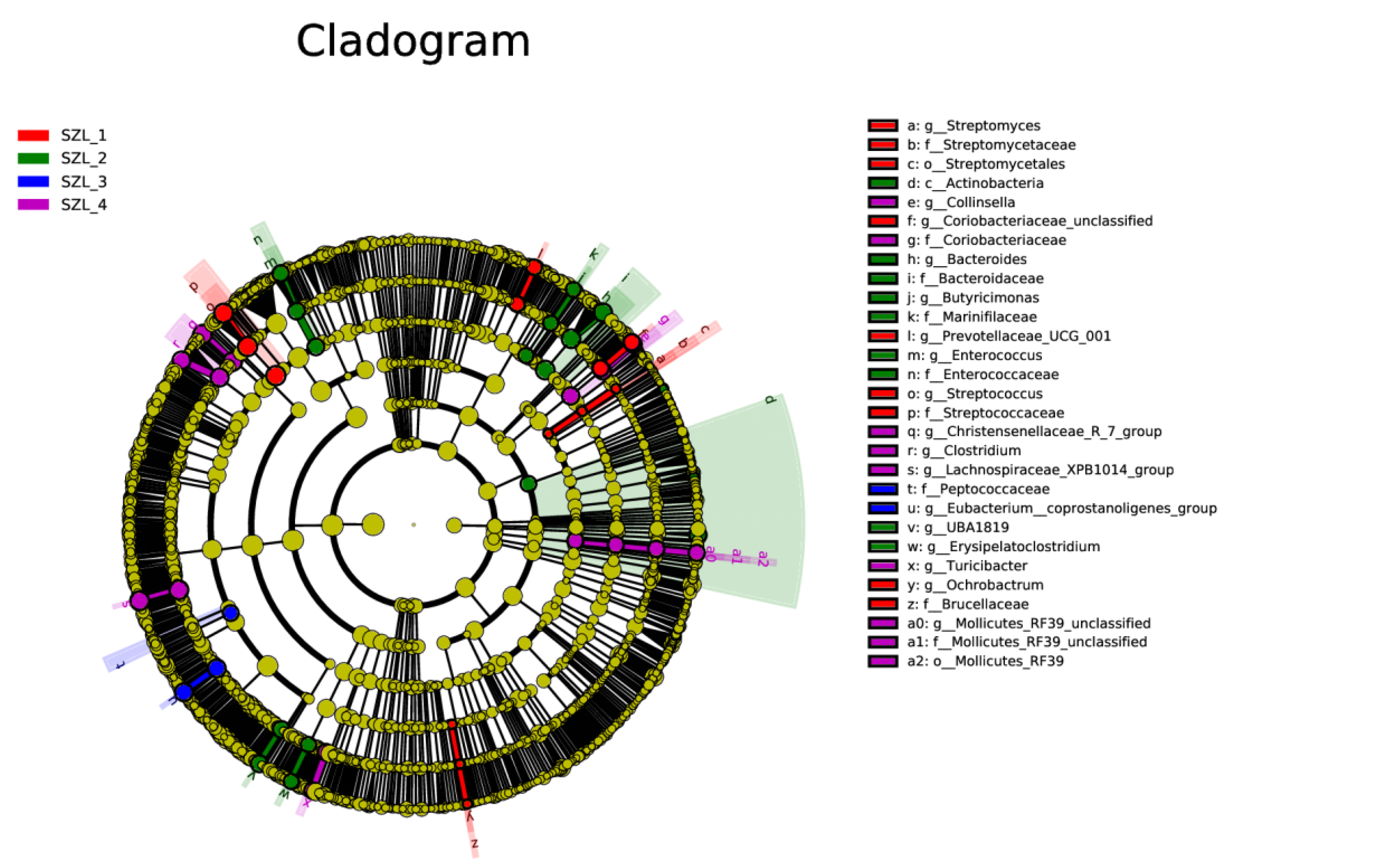

Figure S3. Main cholesterol-degrading bacteria at the genus level. “*” and “**” represent signifificant difference, $p < 0.05$ and $p < 0.01$, respectively.

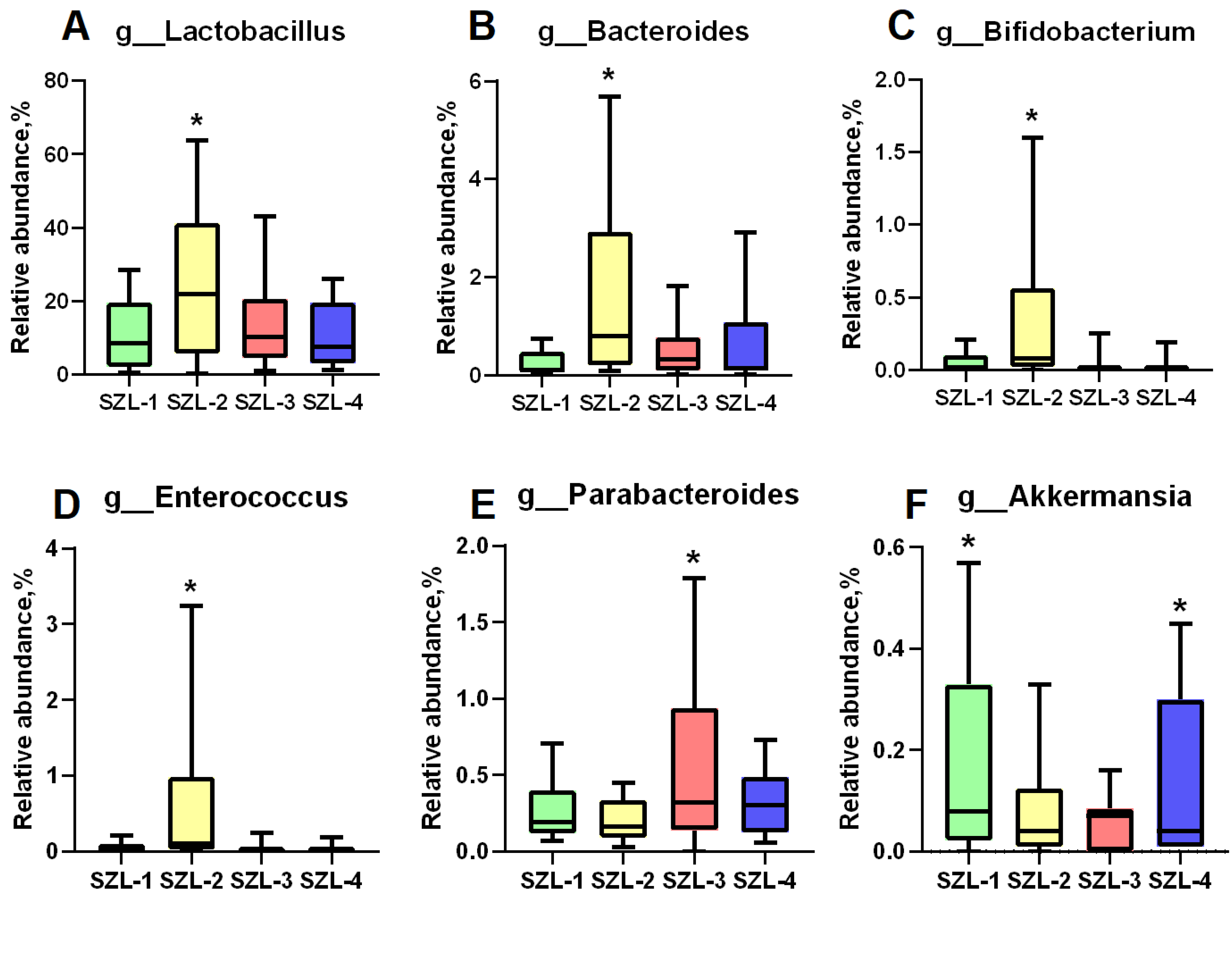

Supplement: Supplementary file 1 [file metabolites-13-00068-s001.zip › supplementary figure.pdf]
